# Supplementary material for: Mosquito Vectors (Diptera: Culicidae) and Mosquito-Borne Diseases in North Africa
Source: Insects. 2022 Oct 20;13(10):962. doi: 10.3390/insects13100962 (PMC9604161; doi:10.3390/insects13100962)
Supplement: Supplementary file 1 [file insects-13-00962-s001.zip › insects-1930544-supplementary.pdf]

Supplementary file

Table S1. Complete list of mosquito species reported in North Africa [18,21].

Mosquito species	Vector status
Aedes aegypti	+++
Aedes albineus	-
Aedes albopictus	+++
Aedes berlandi	-
Aedes biskraensis	-
Aedes caspius	+
Aedes cinereus	-
Aedes coluzzii	-
Aedes detritus	+
Aedes dorsalis	-
Aedes dzeta	-
Aedes echinus	-
Aedes flavescens	-
Aedes geniculatus	-
Aedes mariae	-
Aedes pulcritarsis	-
Aedes pullatus	-
Aedes punctor	-
Aedes quasirusticus	-
Aedes rusticus	-
Aedes vexans	+
Aedes vittatus	-
Aedes zammitii	-
Anopheles ainshamsi	-
Anopheles algeriensis	+
Anopheles arabiensis	+++
Anopheles cinereus s.l.	-
Anopheles claviger s.s.	+
Anopheles coluzzii	+++
Anopheles coustani	+
Anopheles dthali	-
Anopheles hyrcanus	-
Anopheles labranchiae	+++
Anopheles maculipennis s.l.	+++
Anopheles marteri	-
Anopheles multicolor	+++
Anopheles petragnani	-
Anopheles pharoensis	+++
Anopheles plumbeus	-
Anopheles rhodesiensis rupicolus	-
Anopheles rufipesbroussesi	-
Anopheles sacharovi	+
Anopheles sergentii sergentii	+++
Anopheles stephensi	+++
Anopheles superpictus	+
Anopheles tenebrosus	-
Anopheles turkhudi	-
Anopheles ziemanni	-
Coquillettidia buxtoni	-
Coquillettidia richiardii	-

Culex adairi	-
Culex antennatus	+
Culex arbieeni	-
Culex brumpti	-
Culex duttoni	-
Culex deserticola	-
Culex hortensis	-
Culex impudicus	-
Culex laticinctus	-
Culex martinii	-
Culex mimeticus	-
Culex modestus	+
Culex perexiguus	+
Culex pipiens	+++
Culex poicilipes	-
Culex pusillus	+
Culex quinquefasciatus	+++
Culex simpsoni	-
Culex sinaiticus	-
Culex sitiens	-
Culex territans	-
Culex theileri	+
Culex torrentium	-
Culex tritaeniorhynchus	-
Culex univittatus	+
Culex vagans	-
Culiseta annulata	+
Culiseta fumipennis	-
Culiseta litorea	-
Culiseta longiareolata	-
Culiseta morsitans	-
Culiseta subochrea	-
Orthopodomyia pulcripalpis	-
Uranotaenia balfouri	-
Uranotaenia unguiculata	-

+++ Confirmed vector, **+** Potential vector, - No known vector role.
